# *Drosophila* FMRP controls miR-276-mediated regulation of *nejire* mRNA for space-filling dendrite development

**DOI:** 10.1093/g3journal/jkac239

**Published:** 2022-09-14

**Authors:** Hui Li, Elizabeth R Gavis

**Affiliations:** Department of Molecular Biology, Princeton University, Princeton, NJ 08544, USA; Department of Molecular Biology, Princeton University, Princeton, NJ 08544, USA

**Keywords:** Fragile X syndrome, FMRP, *nejire*, dendritic arborization, *Drosophila*, neuron

## Abstract

MicroRNAs are enriched in neurons and play important roles in dendritic spine development and synaptic plasticity. MicroRNA activity is controlled by a wide range of RNA-binding proteins. FMRP, a highly conserved RNA-binding protein, has been linked to microRNA-mediated gene regulation in axonal development and dendritic spine formation. FMRP also participates in dendritic arbor morphogenesis, but whether and how microRNAs contribute to its function in this process remains to be elucidated. Here, using *Drosophila* larval sensory neurons, we show that a FMRP-associated microRNA, miR-276, functions in FMRP-mediated space-filling dendrite morphogenesis. Using EGFP microRNA sensors, we demonstrate that FMRP likely acts by regulating miR-276a RNA targeting rather than by modulating microRNA levels. Supporting this conclusion, miR-276a coimmunoprecipitated with FMRP and this association was dependent on the FMRP KH domains. By testing putative targets of the FMRP–miR-276a regulatory axis, we identified *nejire* as a FMRP-associated mRNA and, using EGFP reporters, showed that the *nejire* 3′ untranslated region is a target of miR-276a in vivo. Genetic analysis places *nejire* downstream of the FMRP–miR-276a pathway in regulating dendrite patterning. Together, our findings support a model in which FMRP facilitates miR-276a-mediated control of *nejire* for proper dendrite space-filling morphology and shed light on microRNA-dependent dendrite developmental pathology of fragile X syndrome.

## Introduction

MicroRNAs (miRNAs) are ∼22-nt small noncoding RNAs that posttranscriptionally regulate gene expression (Iwakawa and Tomari 2015). miRNA biogenesis typically starts with synthesis of primary miRNAs (pri-miRNAs) which are processed by Drosha/DGCR8 to produce short hairpin precursor miRNAs (pre-miRNAs). Pre-miRNAs are exported from the nucleus to the cytoplasm by exportin 5, followed by cleavage by Dicer. One strand from the remaining duplex is subsequently loaded onto Argonaute (Ago), forming a miRNA-induced silencing complex (miRISC), which in turn promotes translational repression and/or degradation of target RNAs by base pairing with complementary sequences in 3′ untranslated regions (3′ UTRs) (Lai 2002; Ha and Kim 2014). miRNAs are highly expressed in the nervous system where they play important roles in neuronal development and function (Kosik 2006; Fineberg *et al.* 2009; Schratt 2009; McNeill and Van Vactor 2012; Rajman and Schratt 2017). They are distributed to dendrites (Sambandan *et al.* 2017) and contribute to spine development and synaptic plasticity by locally regulating protein synthesis (Schratt *et al.* 2006; Siegel *et al.* 2009). Dysfunction of the miRNA pathway has been linked to many neurodevelopmental disorders, such as autism spectrum disorder, Rett syndrome, fragile X syndrome (FXS), and Tourette’s syndrome (Kosik 2006; Fineberg *et al.* 2009; McNeill and Van Vactor 2012; Rajman and Schratt 2017). However, the regulatory roles of miRNAs in the development of complex dendritic arbors are still poorly understood. miRNA biogenesis and function are highly regulated by RNA-binding proteins (RBPs) (Ha and Kim 2014; Connerty *et al.* 2015). For example, TDP-43 interacts with Drosha and pri-miRNAs to facilitate pre-miRNA production (Kawahara and Mieda-Sato 2012). Xrn1, an exonuclease, regulates the turnover of mature miRNAs (Bail *et al.* 2010). In addition, recognition of RNA targets by miRNAs is controlled by a wide variety of RBPs (Kim *et al.* 2021). Pumilio (Kedde *et al.* 2010), IMP2 (Degrauwe *et al.* 2016), FUS (Zhang *et al.* 2018), and Dnd1 (Kedde *et al.* 2007) have been reported to bind to and/or change 3′ UTR RNA structures to promote or suppress miRNA targeting for translational repression.

Fragile X mental retardation protein (FMRP) is a highly conserved RBP that has been implicated in miRNA-mediated gene regulation. FMRP was found to be associated with key components of the miRNA biogenesis pathway, including Ago1 and Dicer (Jin *et al.* 2004), and to bind to several miRNAs, such as *bantam*, *let-7*, miR-125b, miR-132, and miR-181d (Yang *et al.* 2009; Edbauer *et al.* 2010; Wang *et al.* 2015), suggesting that miRNA dysfunction may contribute to FXS. In neuronal development, FMRP was previously reported to regulate translation of synaptic mRNAs by interacting with individual miRNAs and promoting the formation of miRISC for proper synaptic structure and function (Edbauer *et al.* 2010; Muddashetty *et al.* 2011), to mediate axonal transport of miR-181d and local regulation of *map1b* and *calm1* mRNAs for axonal elongation (Wang *et al.* 2015), and to control mature miR-124 levels during dendrite development (Xu *et al.* 2008). Despite the known functions of FMRP in miRNA-mediated synaptic and axonal regulation, whether and how FMRP regulates dendrite patterning through the miRNA pathway remain to be explored.

In this study, using the *Drosophila* larval class IV da (C4da) sensory neurons as a model system, we have identified miR-276 as a regulator of dendritic arbor patterning and field coverage. We further show that miR-276 activity in C4da neuron dendrite development depends on FMRP, which most likely functions in miR-276 RNA targeting rather than by regulating mature miR-276 levels. Consistent with this, FMRP associates with mature miR-276a in a KH domain-dependent manner. By testing previously identified FMRP target transcripts that are also predicted to be miR-276a targets, we discovered potential RNA targets of the FMRP–miR-276a regulatory axis for proper C4da dendritic field coverage. Detailed analysis of one target, *nejire* (*nej*) mRNA, showed that it is enriched in FMRP immunoprecipitates from S2 cells and genetic interaction analyses placed *nej* downstream of FMRP-miR-276 activity. Finally, we show that miR-276 can regulate a *nej* 3′ UTR EGFP reporter when its target site is present. Collectively, these results uncover a mechanism by which FMRP participates in miR-276-mediated regulation of *nej* mRNA to ensure proper space-filling dendrite morphology.

## Materials and methods

### Fly strains

The following transgenic stocks were used: *ppk-GAL4, UAS-CD4::tdGFP* (Bhogal *et al*. 2016); *UAS-mCherry.scramble.sponge* (Bloomington Stock 61501); *ppk-GAL4* (Bloomington Stock 32079); *UAS-mCherry.miR-276a.sponge* (Bloomington Stock 61406); *UAS-mCherry.miR-276b.sponge* (Bloomington Stock 61407); *UAS-mCherry.mir-9c.sponge* (Bloomington Stock 61376); *UAS-mCherry.mir-125.sponge* (Bloomington Stock 61393); *UAS-Fmr1.Z* (Bloomington Stock 6931); *UAS-fmr1-RNAi* (Bloomington Stock 34944; TRiP HMS00248); *UAS-Nf1-RNAi* (Bloomington Stock 53322; TRiP HMC03551) (validated by Moscato *et al.* 2020); *UAS-nej-RNAi* (Bloomington Stock 37489; TRiP HMS01507) (validated by Jia *et al.* 2015); *UAS-inaE-RNAi* (Bloomington Stock 64885; TRiP HMC05758) (validated by Shieh *et al.* 2021); *UAS-Hers-RNAi* (Bloomington Stock 61858; TRiP HMJ23347); *UAS-Ric-RNAi* (Bloomington Stock 82973; TRiP HMC06651); *UAS-Mkp3-RNAi* (Bloomington Stock 57030; TRiP HMS04475); and *UAS-Axn-RNAi* (Bloomington Stock 62434; TRiP HMJ23888) (validated by Nye *et al.* 2020). *ppk-GAL4* was used to drive expression of *UAS* transgenes specifically in C4da neurons. To enhance GAL4/UAS efficiency, the experiments using *UAS-RNAi* lines and *UAS-mCherry-miRNA-SP* lines were performed at 29°C. All other crosses were performed at 25°C.

### Plasmid construction

#### *UAS-pre-miR-276a* transgenes

The *UAS-pre-miR-276a* transgenes were generated using the same strategy as previously described (Xu *et al.* 2008). A 112-bp fragment containing 98 nt of the pre-miR-276a sequence was amplified from genomic DNA with the following pairs of primers: Fwd_pre-miR-276a (5′-GATCCTGAATTCTTTTTTACCTGGTTTTTGCC-3′) and Rev_pre-miR-276a (5′-GGCTATTCTAGAGCATTCACTTGGTTGTTTTTTG-3′). The PCR products and pattB-UASt vector were digested with *EcoRI* and *XbaI* and ligated together to generate pUASt-pre-miR-276a.

#### *pTub-nuc-EGFP-2x-miR-276a* transgenes

A fragment containing 2 copies of perfectly complementary sequence to miR-276a was generated by overlap extension PCR with the following pairs of primers: Fwd_XbaI_2x-miR-276a (5′-GCTATCTAGAAGAGCACGGTATGAAGTTCCTATAACGTTAACGTAACGTTAAAGAGCACGGTATGAAGTTCCTACTCGAGAGGATCGCGC-3′) and Rev_XhoI_2x-miR-276 (5′-GCGCGATCCTCTCGAG-3′). The PCR products and pCaSpeR4_Tub-nuc-EGFP (by courtesy of E. Lai) were digested with *XbaI* and *XhoI* then ligated together to produce pCaSpeR4_Tub-nuc-EGFP_2x-miR-276a. Tub-nuc-EGFP and Tub-nuc-EGFP_2x-miR-276a fragments were amplified from pCaSpeR4_Tub-nuc-EGFP and pCaSpeR4_Tub-nuc-EGFP_2x-miR-276a, respectively, using Fwd_EcoRI_Tub-nuc-EGFP (5′-GATCCTGAATTCGATATCAAGCTTGCACAG-3′) and Rev_BglII_Tub-nuc-EGFP (5′-GACAGTAGATCTGTCGACCTCGACATACATTG-3′) primers. The PCR products were digested with *EcoRI* and *BglII* and ligated individually into the pattB vector digested with *EcoRI* and *BglII* to produce pTub-nuc-EGFP and pTub-nuc-EGFP-2x-miR-276a.

#### *pTub-nuc-EGFP-nej-3′UTR* transgenes

To generate the intact reporter, a 493-bp fragment of the *nej* 3′ UTR was amplified from genomic DNA using Fwd_XbaI_nej-3′UTR (5′-GGCTATTCTAGAGTGCAACAAAATAGCAATAGCC-3′) and Rev_XhoI_nej-3′UTR (5′-GATCCTCTCGAGCGTTTAAGCCTAAAAGTCTATAGC-3′) primers and digested with *XbaI* and *XhoI*. The fragment was then ligated into pCaSpeR4_Tub-nuc-EGFP digested with *XbaI* and *XhoI* to produce pCaSpeR4_Tub-nuc-EGFP_nej-3′UTR. To generate the mut reporter, the miR-276a seed sequence was deleted from the *nej* 3′ UTR fragment by amplifying 2 regions of the *nej* 3′ UTR fragment using Fwd_XbaI_nej-3′UTR (5′-GGCTATTCTAGAGTGCAACAAAATAGCAATAGCC-3′) and Rev_Esp3I_nej-3′UTR_frag1 (5′-ATTACGTCTCAGCGCACAGACACACTCG-3′) and Fwd_Esp3I_nej-3′UTR_frag2 (5′-CCGTCGTCTCGGCGCATTCTTCGATTATTATACATTCATTTAATTTTCGATC-3′) and Rev_XhoI_nej-3′UTR (5′-GATCCTCTCGAGCGTTTAAGCCTAAAAGTCTATAGC-3′) primers. Two PCR products were digested with indicated enzymes and then ligated into pCaSpeR4_Tub-nuc-EGFP digested with *XbaI* and *XhoI* to produce pCaSpeR4_Tub-nuc-EGFP_nej-3′UTR-mut. Tub-nuc-EGFP_nej-3′UTR and Tub-nuc-EGFP_nej-3′UTR-mut were amplified from pCaSpeR4 plasmids using Fwd_EcoRI_Tub-nuc-EGFP (5′-GATCCTGAATTCGATATCAAGCTTGCACAG-3′) and Rev_BglII_Tub-nuc-EGFP (5′-GACAGTAGATCTGTCGACCTCGACATACATTG-3′) primers and digested with *EcoRI* and *BglII* for insertion into the pattB vector to produce pTub-nuc-EGFP_nej-3′UTR and pTub-nuc-EGFP_nej-3′UTR-mut.

### RNA immunoprecipitation and RT-qPCR

*Drosophila* S2 cell culture, transfection, and RNA immunoprecipitation were conducted as previously described (Li and Gavis 2022). FMRP variants were detected by immunoblotting with 1:2,000 DYKDDDDK tag monoclonal antibody (Invitrogen, Cat # MA1-91878) and 1:2,000 HRP sheep anti-mouse antibody (VWR, Cat # 95017-332). Ten nanograms of total RNA was reverse transcribed using the TaqMan MicroRNA Reverse Transcription Kit (Applied Biosystems, Cat # 4366596). Real-time PCR was then performed with TaqMan Fast Advanced Master Mix (Applied Biosystems, Cat # 4444556) and dme-miR-276a TaqMan miRNA Assays (Applied Biosystems, Cat # 4440886). Poly(A) mRNA was reverse transcribed using SuperScript III First-Strand Synthesis System (Invitrogen, Cat # 18080051) and real-time PCR analysis was performed with SYBR Green PCR Master Mix (Thermo Fisher, Cat # 4309155). For −RT controls, nuclease-free water was added instead of reverse transcriptase. *rp49* was used as endogenous control for real-time PCR. The primers listed below were used: chic_Fwd (5′-TGCACTGCATGAAGACAACA-3′) and chic_Rev (5′-GTTTCTCTACCACGGAAGCG-3′); nej_Fwd (5′-GTGGGCACTCAGATGGGTATG-3′) and nej_Rev (5′-CATGCCTGGTATGGCGTTCA-3′); nf1_Fwd (5′-CTTTTGGCACGTTTCGAGGAT-3′) and nf1_Rev (5′-GGTAGCGCGATATGTGGATCA-3′); 14-3-3ζ_Fwd (5′-CGACAGTCGATAAGGAAGAGC-3′) and 14-3-3ζ_Rev (5′-TCTCTGTGACGGACTTCATGG-3′); and rp49_Fwd (5′-CGGATCGATATGCTAAGCTGT-3′) and rp49_Rev (5′-GCGCTTGTTCGATCCGTA-3′).

### Immunofluorescence

Late 3rd instar larva preparation and staining were performed as previously described (Bhogal *et al*. 2016). For better immunostaining efficiency, the larval body wall muscles were removed as described (Tenenbaum and Gavis 2016). FMRP expression was detected with anti-FMRP monoclonal antibody (1:100, Abcam, ab10299) and AlexaFluor 568 goat anti-mouse (1:500, Life technologies, A-11004) secondary antibody. Neuronal membranes were visualized using Alexa 568-conjugated anti-HRP (1:200, Jackson, 123-585-021) and Alexa 647-conjugated anti-HRP (1:200, Jackson, 123-605-021). All antibodies were incubated in blocking buffer containing PBS/0.3% TritonX-100 with 5% normal goat serum either overnight at 4°C (primary antibodies) or for 1.5 h at room temperature (secondary antibodies). Larvae fillets were mounted between a coverslip and slide with VECTASHIELD Antifade Mounting Medium (Vector Laboratory, H-1000-10) and were imaged using a Leica SP5 laser scanning confocal microscope with a 63×/1.4 NA oil objective and sequential scanning. All images are confocal z series projections. Relative nuclear EGFP intensity values were measured by ROI with IntDen function in ImageJ software (https://imagej.nih.gov/ij/) and background was subtracted.

### Analysis of dendrite morphology

C4da (ddaC) neurons from live larvae were mounted individually in 80% glycerol between a slide and a coverslip and imaged by a Leica SP5 laser scanning confocal microscope (40×/1.25 NA oil objective). All images are confocal *z* series projections. For consistency, class IV ddaC neurons from abdominal segments A3–A5 were imaged. At least 10 neurons from 5 or more larvae were imaged and analyzed for each genotype. Quantitative analysis of dendrite morphology was performed with ImageJ software. The dendritic arbor field coverage was quantified by overlaying a grid of 20 × 20 squares on the image of interest and counting the number of empty squares. The dendritic field coverage ratio = # empty squares/400.

### Statistical analysis

All data were analyzed and plotted using GraphPad Prism 9 (https://www.graphpad.com/). Comparisons between 2 groups were performed with the unpaired Student’s *t*-test. For 3 or more groups, 1-way ANOVA with Dunnett’s or Tukey’s multiple comparisons test was used. Values are mean ± SD; ns, not significant; *P < 0.05, **P < 0.01, ***P < 0.001, ****P < 0.0001.

## Results

### miR-276 is required for proper C4da dendritic field coverage

**Fig. 1.**
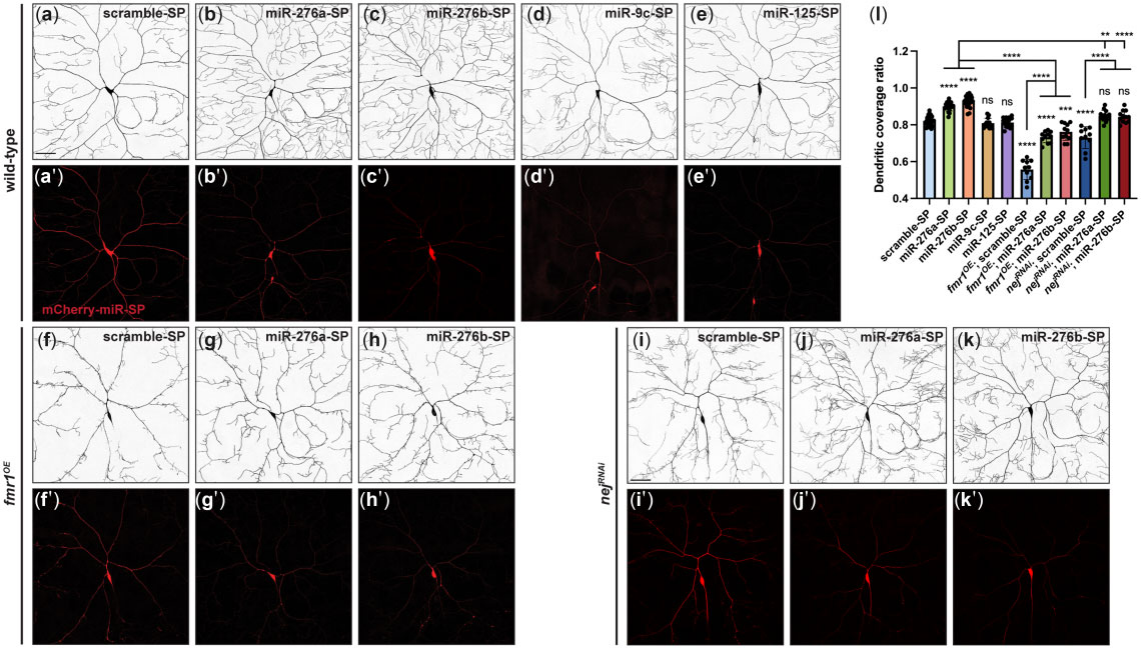
miR-276 genetically interacts with FMRP to regulate C4da dendritic field coverage. Representative images of C4da neurons expressing the mCherry-scramble-sponge (SP) (a–a′), mCherry-miR-276a-sponge (b–b′), mCherry-miR-276b-sponge (c–c′), mCherry-miR-9c-sponge (d–d′), and mCherry-miR-125-sponge (e–e′) driven by *ppk-GAL4*. Representative images of C4da neurons overexpressing *fmr1* (*fmr1^OE^*) together with the mCherry-scramble-sponge (f–f′), mCherry-miR-276a-sponge (g–g′), or mCherry-miR-276b-sponge (h–h′). Representative images of C4da neurons expressing *nej* RNAi together with mCherry-scramble-sponge (i–i′), mCherry-miR-276a-sponge (j–j′), or mCherry-miR-276b-sponge (k–k′). C4da neuronal membranes were labeled with CD4-tdGFP. Expression of the mCherry-miRNA-sponges was confirmed by direct mCherry fluorescence in (a′)–(k′). l) Quantification of the dendritic field coverage ratio in C4da neurons with the indicated genotypes. All images are confocal *z* series projections. Scale bar, 50 μm. Each data point represents a value for one neuron. Values are mean ± SD; ns, not significant; **P < 0.01, ****P < 0.0001, one-way ANOVA with Tukey’s multiple comparisons test.

Several miRNAs identified in FMRP immunoprecipitates from wild-type *Drosophila* ovaries, including miR-9c, miR-125, and miR-276a (Yang *et al.* 2009), have been implicated in the regulation of mammalian dendritic growth and spine formation (Edbauer *et al.* 2010; Giusti *et al.* 2014) and *Drosophila* olfactory memory formation (Li *et al.* 2013). To determine if these potential FMRP-associated miRNAs play a role in dendrite morphogenesis in *Drosophila*, we disrupted their activity in C4da neurons using miRNA sponges, which act as competitive inhibitors by sequestering miRNAs (Fulga *et al.* 2015). Each miRNA sponge was expressed selectively in C4da neurons using *ppk-GAL4* (Fig. 1, a′–e′). As compared to the control scrambled sequence sponge (Fig. 1a), expression of the miR-276a and miR-276b sponges resulted in increased coverage of the dendritic field (Fig. 1, b–e and l), suggesting that miR-276a and/or miR-276b is required to limit the space-filling behavior of C4da dendritic arbors. Notably, this phenotype resembles loss of FMRP (Li and Gavis 2022) (also see Fig. 4, a, i, and n).

### miR-276 genetically interacts with *fmr1* in regulating C4da space-filling dendrite morphogenesis

FMRP has been previously shown to regulate C4da dendrite arborization (Lee *et al.* 2003; Li and Gavis 2022) and to associate with miR-276a in the *Drosophila* ovary (Yang *et al.* 2009). miR-276a and miR-276b, which differ by a single nucleotide at position 10, are members of the *Drosophila* dme-miR-276 family (Supplementary Fig. 1, a and b). We therefore asked if miR-276 is involved in FMRP-mediated dendritic regulation. Overexpression of *fmr1*, which encodes FMRP, in C4da neurons (*fmr1^OE^*) results in a sparse dendritic arbor and dramatically reduced field coverage (Li and Gavis 2022). Expression of either the miR-276a or miR-276b sponge, but not the scrambled sponge, in *fmr1^OE^* neurons partially rescued this dendritic field coverage defect (Fig. 1, f–h and l). Together, these results support the idea that FMRP function in space-filling dendrite morphogenesis is mediated in part by its interaction with miR-276.

The single nucleotide difference between miR-276a and miR-276b falls within the bulge that forms between each miRNA sponge and the bound miRNA (position 9-11, Supplementary Fig. 1c). Thus, miR-276a and miR-276b should each be sequestered by both the miR-276a and miR-276b sponges. miR-276a has functions in regulation of olfactory memory formation in mushroom body neurons (Li *et al.* 2013) and circadian rhythms in the central nervous system (Chen and Rosbash 2016). Because of its known activity in the nervous system, we focused on miR-276a in subsequent experiments, although we cannot rule out the possibility that miR-276a and miR-276b function redundantly in C4da dendrite regulation.

### miR-276a levels are not altered by loss or gain of FMRP in C4da neurons

**Fig. 2.**
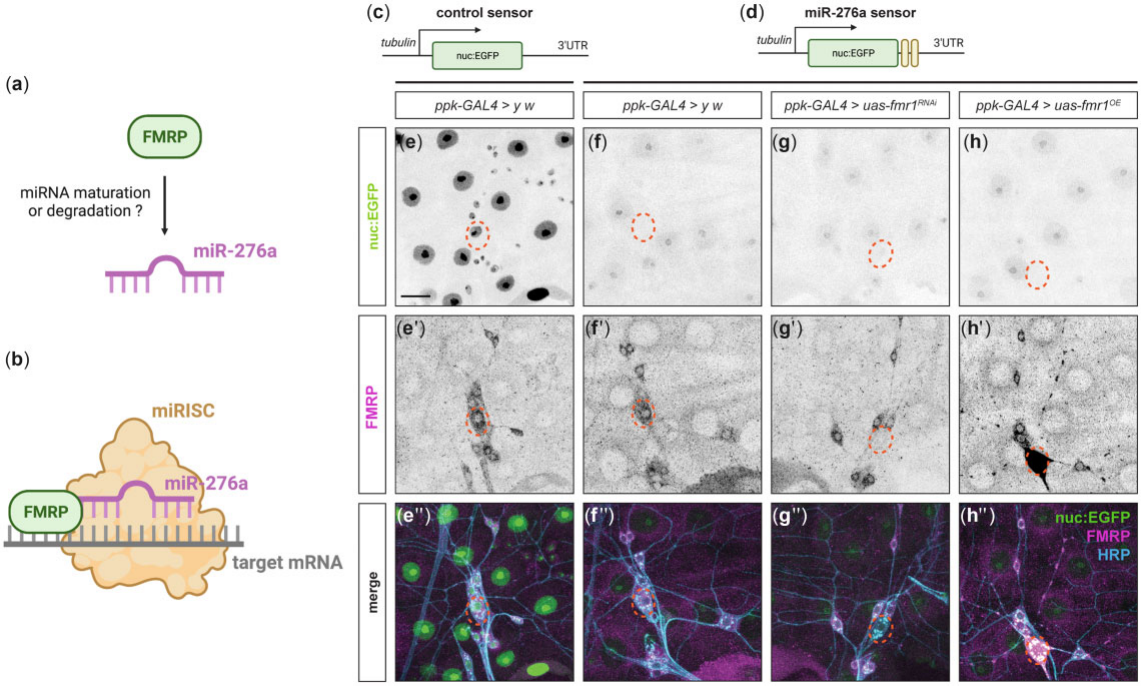
miR-276a levels in C4da neurons are not altered by FMRP. a and b) Two hypotheses for FMRP-mediated dendrite patterning through miR-276a. Schematic illustration of the pTub-nuc:EGFP sensor without (c) or with (d) 2 copies of miR-276a perfect complementary sequences in the SV40 3′ UTR. Nuclear EGFP signals detected in wild-type larvae (e and f), larvae with C4da-specific *fmr1* RNAi (g), or overexpression (OE) (h). FMRP (e′–h′) was detected by anti-FMRP immunofluorescence. Neuronal membranes were visualized by anti-HRP immunofluorescence. e″–h″) Merged images. Dashed circles denote the somas of C4da neurons. All images are confocal *z* series projections. Scale bar, 20 μm. Panels (a-d) were created with BioRender.com.

We next sought to determine how FMRP and miR-276a are mechanistically linked in regulating dendritic patterning. One possibility is that FMRP functions in miRNA maturation and/or stability to regulate steady-state levels of miR-276a (Fig. 2a). Alternatively, FMRP might function in miR-276a RNA targeting (Fig. 2b). To test the first possibility, we generated transgenic flies ubiquitously expressing nuclear EGFP sensors to monitor relative miR-276a levels in vivo (Fig. 2, c and d). EGFP sensors fused to the SV40 3′ UTR, with or without 2 copies of perfectly complementary sequences to miR-276a, were expressed in wild-type larvae or in larvae with C4da neuron-specific *fmr1* RNAi or overexpression. If miR-276a levels are regulated by FMRP, EGFP expression should depend on the level of FMRP. With the control sensor lacking miR-276a complementary sequences, EGFP was detected in the nuclei of epidermal cells and all 4 classes of da neurons (Fig. 2e). By contrast, EGFP expression was dramatically reduced throughout larvae expressing the miR-276a sensor (Fig. 2f), indicating ubiquitous expression of miR-276a at the late 3rd instar larval stage. Expression of the miR-276a sponge, but not the scrambled sponge, in C4da neurons together with the miR-276a sensor significantly restored EGFP expression in the neurons (Supplementary Fig. 2), confirming the effectiveness of both the miR-276a sponge and the miR-276a EGFP sensor. More importantly, decreasing or increasing FMRP levels, as confirmed by anti-FMRP immunofluorescence, did not affect EGFP expression in C4da neurons (Fig. 2, g–h, g′–h′, and g″–h″), indicating that FMRP does not regulate levels of miR-276a.

### FMRP is associated with miR-276a through KH domains

**Fig. 3.**
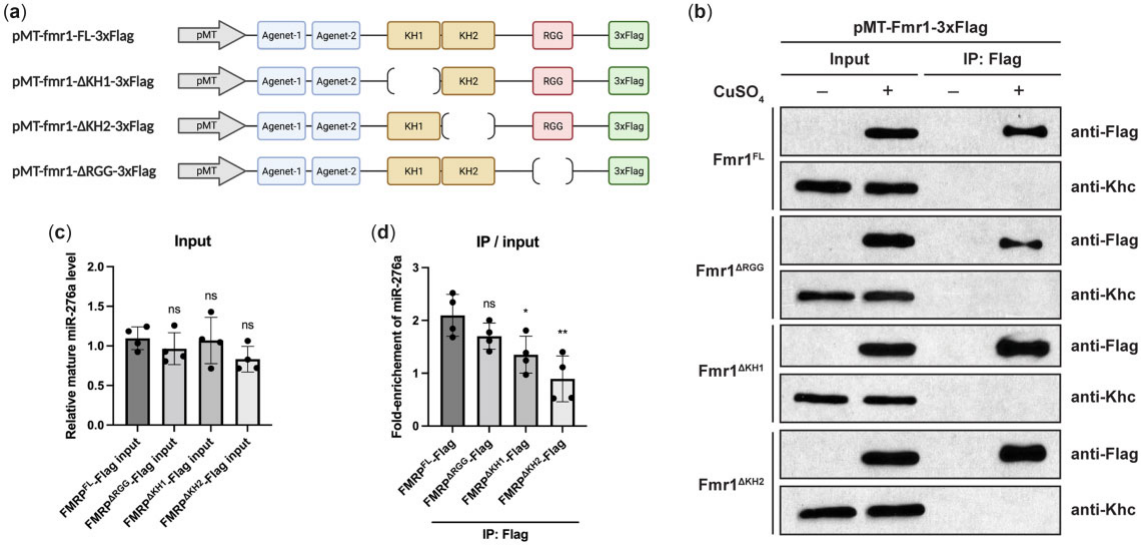
FMRP is associated with miR-276a through KH domains. a) Schematic illustration of constructs for expression of 3xFlag-tagged FMRP and different RBD deleted variants in *Drosophila* S2 cells under control of the metallothionine (MT) promoter. b) Western blotting of lysates from S2 cells transfected with the indicated constructs with (+) or without (−) induction with CuSO_4_. Proteins were detected with anti-DYKDDDDK (Flag) antibody. Uninduced S2 cells were used as controls. Kinesin heavy chain (Khc) was used as the loading control. c) RT-qPCR analysis showing the relative mature miR-276a level in the induced input sample as compared to the corresponding uninduced control input. d) RT-qPCR analysis showing the fold enrichment of mature miR-276a in anti-Flag immunoprecipitates relative to input. Each dot represents one biological replicate with 3 technical replicates averaged. Values are mean ± SD; ns, not significant; *P < 0.05, **P < 0.01, one-way ANOVA with Dunnett’s multiple comparisons test. Panel (a) was created with BioRender.com.

Since FMRP does not control miR-276a levels, we asked whether it might instead function in miR-276a target RNA interaction. We first tested whether FMRP interacts with miR-276a by RNA coimmunoprecipitation. Flag-tagged FMRP (FMRP-3xFlag; Fig. 3a) was expressed in *Drosophila* S2 cells and immunoprecipitated with anti-DYKDDDDK antibody (Fig. 3b). RT-qPCR analysis of RNA extracted from the immunoprecipitates showed that amount of mature miR-276a was similar in S2 cells with or without induction of FMRP-3xFlag expression (Fig. 3c), which is consistent with results from the EGFP sensor experiments showing that miR-276a levels were unaffected by overexpression of FMRP in C4da neurons (Fig. 2, f and h). Mature miR-276a was enriched 2-fold in the FMRP-3xFlag immunoprecipitate compared to the control (Fig. 3d), indicating that FMRP associates with miR-276a in vivo, either directly or indirectly (see *Discussion*).

FMRP has 3 RNA-binding domains (RBDs)—KH1, KH2, and RGG. The KH domains were previously shown to facilitate miRNA: mRNA complex formation in vitro (Plante *et al.* 2006). To assess the involvement of FMRP’s RBDs in its association with miR-276a, we generated a set of constructs to express Flag-tagged FMRP variants with individual RBD deleted in S2 cells (Fig. 3a). Following immunoprecipitation of the FMRP variants (Fig. 3b), the amount of coimmunoprecipitated miR-276a was quantified by RT-qPCR. Deletion of either the KH1 or the KH2 domain resulted in the loss of miR-276a enrichment in FMRP immunoprecipitates (Fig. 3d), indicating that both KH domains are indispensable for FMRP to bind to miR-276a.

### Predicted FMRP–miR-276a regulatory targets *nej*, *Ric*, and *Mkp3* are required for C4da dendrite arborization

**Fig. 4.**
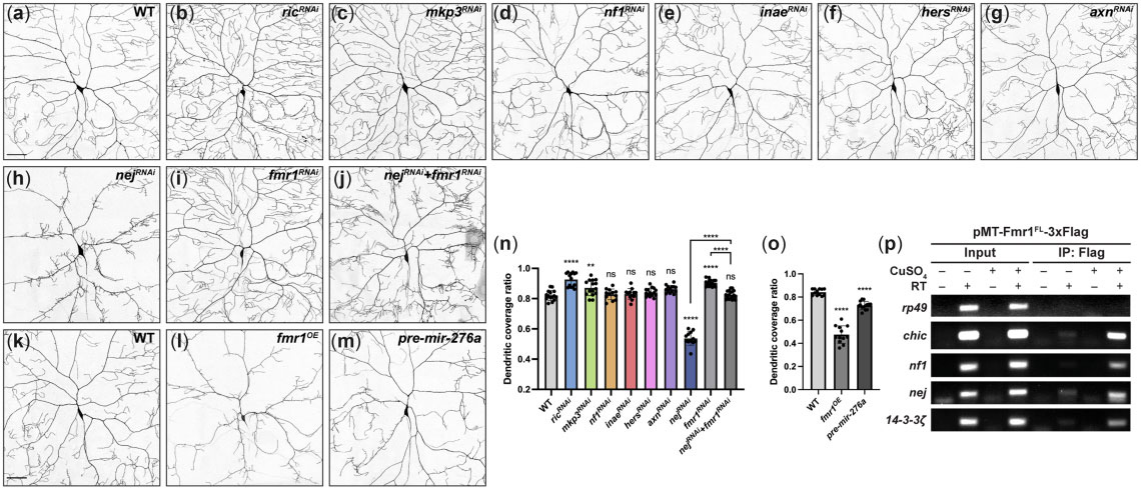
*nej* is involved in FMRP-mediated dendritic field coverage. Representative images of wild-type (WT) (a), *Ric^RNAi^* (b), *Mkp3^RNAi^* (c), *Nf1^RNAi^* (d), *inaE^RNAi^* (e), *Hers^RNAi^* (f), *Axn^RNAi^* (g), *nej^RNAi^* (h), *fmr1^RNAi^* (i), and *nej^RNAi^* + *fmr1^RNAi^* (j) C4da neurons. Representative images of WT (k), *fmr1^OE^* (l), and pre-miR-276a expressing (m) C4da neurons. Membranes were labeled with CD4-tdGFP. All images are confocal *z* series projections. Scale bar, 50 μm. n–o) Quantification of the dendritic field coverage ratio for C4da neurons with the indicated genotypes. Each data point represents a value for one neuron. Values are mean ± SD; ns, not significant; **P < 0.01, ****P < 0.0001, one-way ANOVA with Tukey’s multiple comparisons test. p) RT-PCR gel analysis of *Nf1*, *nej*, and *14-3-3ζ* mRNAs in FMRP-3xFlag immunoprecipitates. For −RT controls, nuclease-free water was added instead of reverse transcriptase. *chic* was used as a positive control (Li and Gavis 2022); *rp49* was used as a negative control.

To identify potential targets of the FMRP–miR-276a regulatory axis, we focused on the overlap between previously identified FMRP targets (Darnell *et al.* 2011; Ascano *et al.* 2012; Maurin *et al.* 2018) and predicted miR-276 targets (TargetScan 7.2), which includes *nej*, *Neurofibromin 1* (*Nf1*), *Mitogen-activated protein kinase phosphatase 3* (*Mkp3*), *Ras-related protein interacting with calmodulin* (*Ric*), *inactivation no afterpotential E* (*inaE*), *Histone gene-specific Epigenetic Repressor in late S phase* (*Hers*), and *Axin* (*Axn*). To determine if these putative targets function in C4da dendrite arborization, we specifically knocked them down in C4da neurons using RNAi driven by *ppk-GAL4*. miRNAs typically downregulate their targets by promoting mRNA degradation and/or translational repression (Iwakawa and Tomari 2015). However, because miRNAs often have only modest regulatory effects, we expected that depleting targets of FMRP–miR-276a regulatory pathway would at least partially mimic *fmr1* overexpression in C4da neurons. Knockdown of *Nf1*, *inaE*, *Hers*, and *Axn* had no obvious phenotypic consequences (Fig. 4, a, d–g, and n). *Ric* and *Mkp3* RNAi each resulted in increased dendritic field coverage, which resembles *fmr1* knockdown rather than overexpression (Fig. 4, a–c, i, and n). On the contrary, *nej* RNAi led to a dramatic decrease in the dendritic coverage ratio, to an extent comparable to *fmr1^OE^* neurons (Fig. 4, h, l, and n). *nej* encodes *Drosophila* CREB-binding protein, which acts as a transcriptional coactivator and acetylates histones to regulate gene expression (Akimaru *et al.* 1997; Das *et al.* 2009). Overexpression of precursor miR-276a (pre-miR-276a) in C4da neurons resulted in similar dendritic coverage defects (Fig. 4, k–m and o), suggesting that *nej* might act in C4da dendritic morphogenesis through the FMRP–miR-276a regulatory pathway.

### *nej* interacts with FMRP in regulating C4da dendrite patterning

We further tested if *nej* is controlled by FMRP using genetic and biochemical analyses. Double RNAi of *fmr1* and *nej* rescued both the increased dendritic field coverage caused by *fmr1* RNAi and the decreased dendritic field coverage caused by *nej* RNAi (Fig. 4, h–j and n), suggesting that *nej* genetically interacts with *fmr1* in regulating C4da space-filling dendrite arborization. To determine if *nej* physically interacts with FMRP, we performed RT-PCR for RNAs that coimmunoprecipitated with FMRP-Flag from S2 cells. *nej*, as well as other 2 putative RNA targets, *Nf1* and *14-3-3ζ*, coimmunoprecipitated with FMRP (Fig. 4p). Along with the phenotypic analysis above, our results suggest that *nej* mRNA is a target of FMRP in C4da dendrite patterning.

### The *nej* 3′ UTR is a target of miR-276a in C4da neurons

**Fig. 5.**
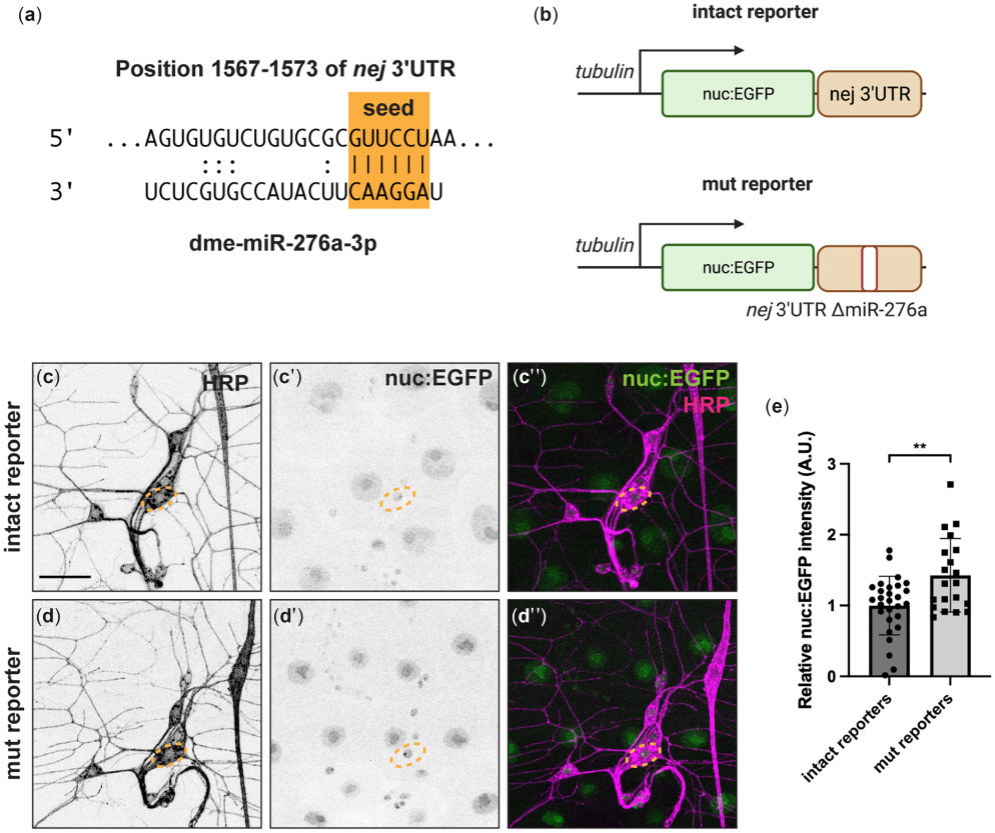
*nej* 3′ UTR is a target of miR-276a and FMRP in C4da neurons. a) Predicted *miR-276a* recognition element in the *nej* 3′ UTR. b) Schematic illustration of EGFP reporters containing a 493 bp fragment of the *nej* 3′ UTR with (intact) or without (mut) predicted miR-276a recognition elements sequence. Nuclear EGFP (c′–d′) from the indicated *nej* 3′ UTR EGFP reporters was detected directly in wild-type larvae and neuronal membranes (c–d, magenta) were visualized using anti-HRP immunofluorescence. c″–d″) Merged images. Dashed circles denote the somas of C4da neurons. All images are confocal z series projections. Scale bar, 30 μm. e) Quantification of relative nuclear EGFP fluorescence intensity from the indicated genotypes. Each data point represents a value of one neuron. Values are mean ± SD; ns, not significant; **P < 0.01, ****P < 0.0001, unpaired Student’s *t*-test. Panels (a) and (b) were created with BioRender.com.

*nej* has a predicted miR-276a recognition element in its 3′ UTR (positions 1,567–1,573; TargetScan7.2) (Fig. 5a). To confirm that *nej* 3′ UTR is a target of miR-276a in C4da neurons, we generated a ubiquitously expressed nuclear EGFP reporter with a 493-bp fragment from the *nej* 3′ UTR containing the predicted miR-276a recognition site (intact reporter) and a corresponding reporter with the recognition site deleted (mut reporter) (Fig. 5b) inserted in the SV40 3′ UTR. EGFP expression from the mut reporter was significantly increased relative to that from the intact reporter (Fig. 5, c–e), consistent with the idea that the reporter expression is regulated by miR-276a. However, EGFP expression from the mut reporter was much lower than that of the EGFP control sensor with only the SV40 3′ UTR (Fig. 2, a and c), indicating that other factors may target this 493-bp region to control *nej* expression.

As a further test that *nej* acts downstream of miR-276a, we knocked down *nej* by RNAi in C4da neurons expressing either of the miR-276 sponges. The increase in dendritic field coverage observed for the sponges was rescued by *nej* RNAi, consistent with a role for miR-276 in downregulating *nej* (Fig. 1, i–l). In sum, our results provide evidence that regulation of *nej* by binding of miR-276 to its 3′ UTR is necessary for proper C4da dendritic field coverage.

## Discussion

miRNAs contribute to the regulation of synaptic structure and axon elongation by FMRP, a highly conserved RBP that functions in different aspects of neuronal development (Edbauer *et al.* 2010; Muddashetty *et al.* 2011; Wang *et al.* 2015). However, their roles in FMRP-mediated dendrite patterning remained unclear. Here, we uncover a role for a FMRP-associated miRNA, miR-276, in FMRP-dependent regulation of space-filling dendrite morphology. FMRP has been shown to regulate the steady-state levels of miRNAs including miR-124 in *Drosophila* larvae (Xu *et al.* 2008). However, our results are most consistent with a role for FMRP in regulating RNA targeting by miR-276 rather than mature miR-276a levels, indicating multiple regulatory roles of FMRP in miRNA-mediated gene expression control of neuronal development. Given the wide range of RNAs identified as FMRP targets (Darnell *et al.* 2011; Ascano *et al.* 2012; Maurin *et al.* 2018), association with miR-276a might contribute to FMRP’s target selectivity and/or provide yet another point of control in addition to translation initiation (Napoli *et al.* 2008) and elongation (Darnell *et al.* 2011; Chen *et al.* 2014).

Our findings support a model in which FMRP facilitates miR-276a targeting of *nej* mRNA for posttranscriptional regulation. *nej*-associated FMRP may help recruit miRISC to the transcript for regulation of gene expression. Deletion of the KH domains significantly alleviated FMRP-mediated enhancement of miRNA:mRNA complex formation in vitro (Plante *et al.* 2006). Consistent with this, mature miR-276a coimmunoprecipitated with FMRP from *Drosophila* S2 cells and this interaction was dependent on both the KH1 and KH2 domains, suggesting that FMRP may interact directly with miR-276a or the miR-276a-*nej* complex through the KH domains. Alternatively, since FMRP was found to coimmunoprecipitate with Ago (Jin *et al.* 2004), it might function in miR-276a targeting via KH-dependent interactions with protein components of miRISC. Lastly, FMRP could indirectly facilitate miR-276a binding to target mRNAs. For example, FMRP might function together with other proteins, such as the RNA helicase MOV10, to help unwind RNA secondary structure and expose miRNA-recognition elements for miRISC targeting (Kenny *et al.* 2014). In addition to their synergistic effects, miR-276a and FMRP might independently contribute to the regulation of dendrite space-filling morphogenesis. A previous study demonstrated that phosphorylation of FMRP inhibits miR-125a-mediated translational regulation of *PSD-95* mRNA (Muddashetty *et al.* 2011); therefore, as an added layer of complexity, posttranslational modifications could affect FMRP’s functions in miRNA-mediated gene regulation.

Since individual miRNAs often have modest regulatory effects on their targets (Baek *et al.* 2008; Selbach *et al.* 2008), and removal of the miR-276a-recognition element from the *nej* 3′ UTR did not completely restore expression of the EGFP reporter, it is likely that other factors also contribute to regulation of *nej* expression. For example, the 493-bp *nej* 3′ UTR fragment used in our analysis contains a predicted recognition element for dme-miR-2/5/6/11/13/308 (positions 1,651–1,657; TargetScan 7.2), indicating an involvement of these miRNAs in *nej* 3′ UTR regulation. Moreover, given the critical roles of RBPs in RNA posttranscriptional regulation (Glisovic *et al.* 2008), they are also likely to contribute to 3′ UTR-mediated regulation of *nej*.

Interestingly, knockdown of 2 FMRP target RNAs—*Ric* and *Mkp3—*that are also predicted to be miR-276a targets led to increased dendritic field coverage, similar to that of *fmr1* knockdown neurons. This effect could be explained by an overlap in the binding sites for FMRP and miR-276a, resulting in competition between FMRP and miRISC for the target RNAs (Kim *et al.* 2021). Thus, FMRP might function to prevent *Ric* and *Mkp3* mRNAs from being targeted by miR-276a-induced silencing complex, thereby upregulating their expression. Whether these RNAs are controlled by FMRP and/or miR-276a or act independently in dendrite morphogenesis warrants further study.

## Supplementary Material

jkac239_Supplementary_Data

## Data Availability

Plasmids and transgenic flies are available upon request. The data underlying this article are available within the article and in the Supplementary material. Supplemental material is available at G3 online.
