# Supplementary material for: Drosophila FMRP controls miR-276-mediated regulation of nejire mRNA for space-filling dendrite development
Source: G3 (Bethesda). 2022 Sep 14;12(11):jkac239. doi: 10.1093/g3journal/jkac239 (PMC9635640; doi:10.1093/g3journal/jkac239)
Supplement: jkac239_Supplementary_Data [file jkac239_supplementary_data.docx]

**Supplemental Figure Legends**

**Figure S1. Genetic characterization of dme-miR-276a and dme-miR-276b**

(A) Gene locus of dme-miR-276a and dme-miR-276b. (B) Sequence alignment of mature miR-276a and miR-276b. The single nucleotide difference is indicated in red. (C) Base pairing of miR-276a-sponge and miR-276b-sponge to mature miR-276a. Created with BioRender.com.

**Figure S2. Expression of miR-276a sponge restored expression of miR-276a EGFP sensor**

(A-B) Representative images of pTub-nuc:EGFP miR-276a sensors in larvae expressing the mCherry-scramble-sponge (A-A') and mCherry-miR-276a-sponge (B-B') in C4da neurons using *ppk-GAL4*. A'-B' show merged images. All images are confocal z series projections. Scale bar, 30 μm. (C-D) EGFP intensity profiles along the orange lines in A-B. (E) Quantification of relative nuclear EGFP intensity from the indicated genotypes. Values are mean ± SD; ****p < 0.0001, unpaired student's t test.
